# *Lactobacillus reuteri* FN041 Alleviates Radiation Enteritis by Regulating M2 Macrophages Polarization Based on Gut Microbiota and Its Metabolites

**DOI:** 10.1002/fsn3.70871

**Published:** 2025-08-27

**Authors:** Suisui Jiang, Sai Yao, Chunyun Lu, Ying Zhou, Tianlin Gao, Shichao Liu, Feng Zhong

**Affiliations:** ^1^ Institute of Nutrition and Health, School of Public Health Qingdao University Qingdao Shandong China; ^2^ Qingdao Central Hospital University of Health and Rehabilitation Sciences Qingdao Shandong China

**Keywords:** fatty acid oxidation, gut microbiota, inflammation, M2 macrophages polarization, radiation enteritis

## Abstract

Radiotherapy, a common method for treating pelvic and abdominal cancer, can easily result in radiation enteritis (RE). The gut microbiota and its metabolites have a crucial role in regulating macrophage polarization and maintaining immune homeostasis. *Lactobacillus reuteri* FN041, derived from human milk, can promote M2 polarization while attenuating RE. After FN041 treatment, the crypt depth and goblet cell numbers were significantly improved in RE mice. The levels of Muc2, ZO‐1, and Occludin were significantly higher in the FN041 group than in the RE group. FN041 stimulated the polarization of macrophages toward M2 and inhibited the expression of IL‐6 and TNF‐α in RE mice. Mechanically, FN041 regulated the gut microbiota and metabolites, including key intermediates in phospholipid synthesis such as CDP‐DG, prostaglandins, and their phospholipid derivatives (including PI, PE, PA, and PS), which were linked to M2 macrophage polarization. Collectively, FN041 exerts a protective effect against RE by modulating macrophage polarization based on gut microbiota and its metabolites.

## Graphical abstract

*Lactobacillus reuteri* FN041 treatment improved colon histology, physical barrier integrity, and mucus integrity. Collectively, FN041 exerts a protective effect against radiation enteritis by modulating macrophage polarization based on gut microbiota and its metabolites.

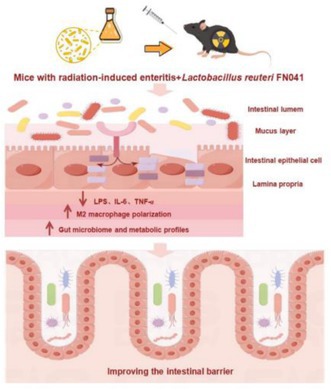

## 1 Introduction

Radiation enteritis (RE) is an intestinal complication resulting from irradiation for abdominal and pelvic cancer patients, and its development process is very complicated (Moraitis et al. 2023). RE may present clinically with frequent stools, diarrhea, and blood (Harb et al. 2014). The incidence rate of acute intestinal toxicity symptoms is 50% and is still increasing year by year (Hauer‐Jensen et al. 2014; Loge et al. 2020). Radiotherapy damages tumor cells and also damages normal gastrointestinal cells, resulting in RE. The impaired intestinal barrier could be attacked by intestinal microbiota, resulting in the release of metabolites such as LPS into the bloodstream (Gerassy‐Vainberg et al. 2018). This can trigger systemic inflammation, which can affect the patient's immunity and reduce their quality of life (Fan et al. 2022). Moreover, severe RE might increase the mortality of cancer patients.

Diverse microorganisms exhibit significant variations in sensitivity to ionizing radiation (Zhao et al. 2021). High‐energy rays can directly damage the DNA, proteins, and cell membrane structures of microorganisms. Probiotics typically lack robust DNA repair mechanisms or antioxidant defense systems present in certain pathogenic bacteria, making them more susceptible to being killed or severely inhibited in growth (Ciorba et al. 2012). Pathogenic bacteria, such as certain strains of *Escherichia coli* and *Staphylococcus aureus* , possess more efficient DNA repair enzymes and thicker cell wall structures. These structures exhibit higher survival rates under equivalent radiation doses (Hing et al. 2022). It has been reported that radiation exposure induces microbiota dysbiosis by reducing the relative abundance of probiotics (*Lactobacillus* spp. and *Bifidobacterium* spp.) and enhancing the relative abundance of pathogenic microbiota ( *Escherichia coli* and *Staphylococcus* spp.) (Jian et al. 2021). The conditional pathogens would reproduce while the relative abundance of probiotics reduces (Dong et al. 2025). It has been reported that gram‐negative bacteria, particularly *Escherichia coli* and *Pseudomonas*, dominate the gut microbiota that breaches the compromised barrier in RE mice (Wang et al. 2019; Lapointe et al. 2009). That suppresses the growth of probiotics and promotes endotoxin secretion. The gut microbiota dysbiosis exacerbated RE and disrupted the normal function of the gut barrier. Probiotics can maintain intestinal homeostasis and intestinal barrier function, whereas harmful bacteria can damage the integrity of the intestinal barrier. *Lactobacillus* spp. supplementation enhanced the gut barrier by regulating the expression of ZO‐1 and occludin (Karczewski et al. 2010; Li, Han, et al. 2021). In addition, short‐chain fatty acids derived from probiotics increased the expression of claudin‐3 and occludin by G protein‐coupled receptors. *Escherichia coli* affects the expression of intestinal barrier‐related proteins, such as claudin‐1, occludin, ZO‐1, and JAM‐1 (Qin et al. 2009). Impaired intestinal barrier is unable to prevent the invasion of pathogenic bacteria, which could activate the NF‐kB signaling pathway and generate pro‐inflammatory cytokines.

Moreover, gut microbiota directly or indirectly influences the host immune response and tissue repair, which play a crucial role in maintaining intestinal health (Schluter et al. 2020; Yang et al. 2023). *Lactobacillus acidophilus* LA‐5 plus *Bifidobacterium animalis* subsp. *lactis* BB‐12 treatment reduced the incidence and severity of RE (Linn et al. 2019). Probiotics ( *Lactobacillus rhamnosus* GG plus BB‐12) showed significant changes in histopathological findings and inflammation cytokines (Kaymakci et al. 2024). *Lactobacillus intestinalis* suppressed Th17 cells in RE mice by inhibiting the secretion of serum amyloid A and SAA2 (Wang et al. 2023). *Lactobacillus paracasei* significantly increases the number of CD11c^+^PDCA‐1^+^ plasmacytoid dendritic cells and Ly6Chi monocytes, promoting host defense (Kim et al. 2023). *Lactobacillus johnsonii* mediates intestinal barrier homeostasis by promoting M2 macrophage polarization to attenuate diarrheal disease (Tao et al. 2025). The exopolysaccharide of *Bacillus subtilis* markedly enhanced the expression of the M2 macrophage marker, which prevented T cell‐mediated disease (Paynich et al. 2017). Short‐chain fatty acids, one kind of beneficial products of the intestinal flora, recruit neutrophils to the inflammation site through activation of the NLRP3 inflammasome (Le Poul et al. 2003). Fatty acid oxidation is closely related to the M2 state, further mediating tissue repair (Yan and Horng 2020). Based on the above findings, gut microbiome homeostasis is essential to maintain a healthy gut.

Currently, the clinical treatment used for relieving RE mainly includes basic supportive therapy, symptom‐guided medication, hyperbaric oxygen therapy, endoscopic, and surgical intervention (Chen et al. 2024). By now, there is no medical cure, and all treatments are only palliative. Probiotics play a beneficial role in improving the balance of the intestinal microbiome and enhancing the immunity of the host (Lee et al. 2024; Tang et al. 2025). At present, only a few probiotics are effective in improving diarrhea symptoms in acute radiation‐induced bowel injury, and its influencing mechanism is still unclear. *Lactobacillus reuteri* FN041 is an indigenous bacterium derived from human milk that could improve the structure of gut microbiota and immune response (Zhao et al. 2022). The purpose of this paper was to investigate whether FN041 could modulate intestinal barrier damage induced by irradiation and its potential mechanisms.

## 2 Materials and Methods

### 2.1 Animal Experiments

Thirty‐two 8‐week‐old male C57BL/6 mice were obtained from Beijing Vital River Laboratory Animal Technology Co. Ltd. (Beijing, China). After 1 week of acclimatization, they were randomly divided into four groups.

After a 1‐week adaptation period, mice were randomly divided into four groups, as shown below: (1) the control group (CN), mice without irradiation received a phosphate buffered solution (200 μL/day) by intragastric gavage; (2) the model group (RE), along with intragastric gavage of phosphate buffered solution (200 μL/day), were subjected to irradiation on the 8th day; (3) the *Lactobacillus rhamnosus* GG group (LGG), received 10^9^ CFU/day for 7 days before irradiation and were exposed to irradiation on the 8th day; (4) the *Lactobacillus reuteri* FN041 group (FN041), received 10^9^ CFU/day for 7 days before irradiation and were exposed to irradiation on the 8th day. The mice in the RE, LGG, and FN041 groups were exposed to 12 Gy of total body irradiation with a dose rate of 5.0 Gy/min using an x‐ray radiation source (Clinac 2100EX; Varian Medical Systems). The irradiation dosage refers to the method of Xu, Wang, et al. (2024). Mice body weight was monitored daily after radiation and euthanized on Day 3.5. The colon tissue and blood were collected for further use. All procedures were performed in accordance with the guidelines in the Care and Use of Animals and with the approval of the Ethical Committee of Experimental Animal Welfare of Qingdao University (no. 20200924C575020210118046).

### 2.2 Measurement of Biochemical Indices in Serum and Tissues

The levels of TNF‐α, IL‐6, and IL‐10 in serum were measured using commercial kits (Nanjing Jiancheng Technology Co. Ltd., Nanjing, Jiangsu, China). Lipopolysaccharides in serum were determined by an ELISA kit (JM‐02652M1; Jiangsu Jingmei Biological Technology Co. Ltd., Jiangsu, China).

### 2.3 Histological Staining and Immunohistochemistry

Colon tissues were paraffin‐embedded and sliced into 5‐μm sections. They were then stained with hematoxylin and eosin, which were used to observe the colon morphology. The damage scores for inflammatory, epithelial, and total score were assayed using the method of Jilling et al. (2004). Staining for macrophages was performed on 7‐μm paraffin‐embedded sections. After blocking with horse serum, the primary antibody rat anti‐mouse CD206 (91992T, 1:500; Cell Signaling Technology Inc., USA) was applied and incubated for 60 min. It was then incubated with the secondary antibody, biotinylated anti‐rat IgG. Macrophage staining was determined using ImageJ.

For immunofluorescence staining, the primary antibodies used were rat anti‐mouse Occludin (91131, 1:200; Cell Signaling Technology Inc.) and ZO‐1 (13663, 1:200; Cell Signaling Technology Inc.).

### 2.4 Alcain Blue Staining

Fresh colon tissues were immediately placed into Carnoy's fluid and then embedded in paraffin. After dewaxing, the embedded colon was dyed for10–20 min using alcian blue and nuclear‐fast red, and then washed with distilled water. Then, the tissues were stained with a solid red nuclear stain for 10 min and washed with distilled water. Samples were observed by microscopy to analyze the mucus layer.

### 2.5 16S rDNA Sequencing Analysis

The mice were euthanized 3.5 days postirradiation, and cecal contents were collected. The structure of the gut microbiome was measured using the previous method according to Xu, Lu, et al. (2024). DNA in feces was extracted according to the instructions (Genomic DNA kit; Omega Biotek, GA, USA). Bacterial 16S rDNA sequencing was analyzed according to the instructions (Beijing Genomic Institution, Wuhan, China).

### 2.6 Measurement of Short‐Chain Fatty Acids

Cecal contents (10 mg) were dissolved in distilled water (400 μL) to prepare a sample solution. The supernatant was collected after centrifuging at 12,000 *g* for 10 min. Then, 50 μL of the supernatant was mixed with 250 μL of distilled water, 30 μL of 50% hydrochloric acid, and 3.3 mL of ethyl ether. Meanwhile, DL‐2‐methyl butyric acid was dissolved in 50% hydrochloric acid as an internal standard. Samples were incubated for 5 min and centrifuged at 12,000 *g* for 5 min at 4°C. The collected supernatant was measured using GC–MS (Agilent 8890 GC system) with an Agilent HP‐5 capillary column (30 m × 0.25 mm × 0.25 μm). The samples were tested with a starting temperature of 60°C for 5 min, followed by increasing the temperature to 110°C with an increment of 10°C/min. Then, the temperature was increased to 250°C with an increment of 35°C/min and maintained for 1 min. All samples were run with helium as the carrier gas at a flow rate of 1.0 mL/min.

### 2.7 Analysis of Serum Metabolites

Serum metabolites in all groups were measured using Agilent Technologies 6530C Q‐TOF LC/MS equipped with the column (ACQUITY UPLC BEH C18 Column 2.1 × 100 mm, 1.7 μm). Serum (20 μL) was mixed with acetonitrile (300 μL) and centrifuged at 12,000 *g* for 15 min. Then, the supernatant was collected and filtered using a 0.22‐μm membrane filter. The mobile phases were 0.1% formic acid (mobile phase A) and acetonitrile (mobile phase B). The column temperature was kept at 40°C, and the flow rate was 0.5 mL/min. The injected volume was 2 μL.

### 2.8 Statistical Analysis

All experimental data were analyzed using a one‐way ANOVA followed by Duncan's post hoc test (IBM SPSS Statistics 20). The results were expressed as mean ± standard deviations, and significance was set as *p < 0.05, **p < 0.01, and ***p < 0.001.

## 3 Results

### 3.1 *Lactobacillus reuteri* FN041 Supplementation Attenuated Radiation Enteritis

**FIGURE 1:**
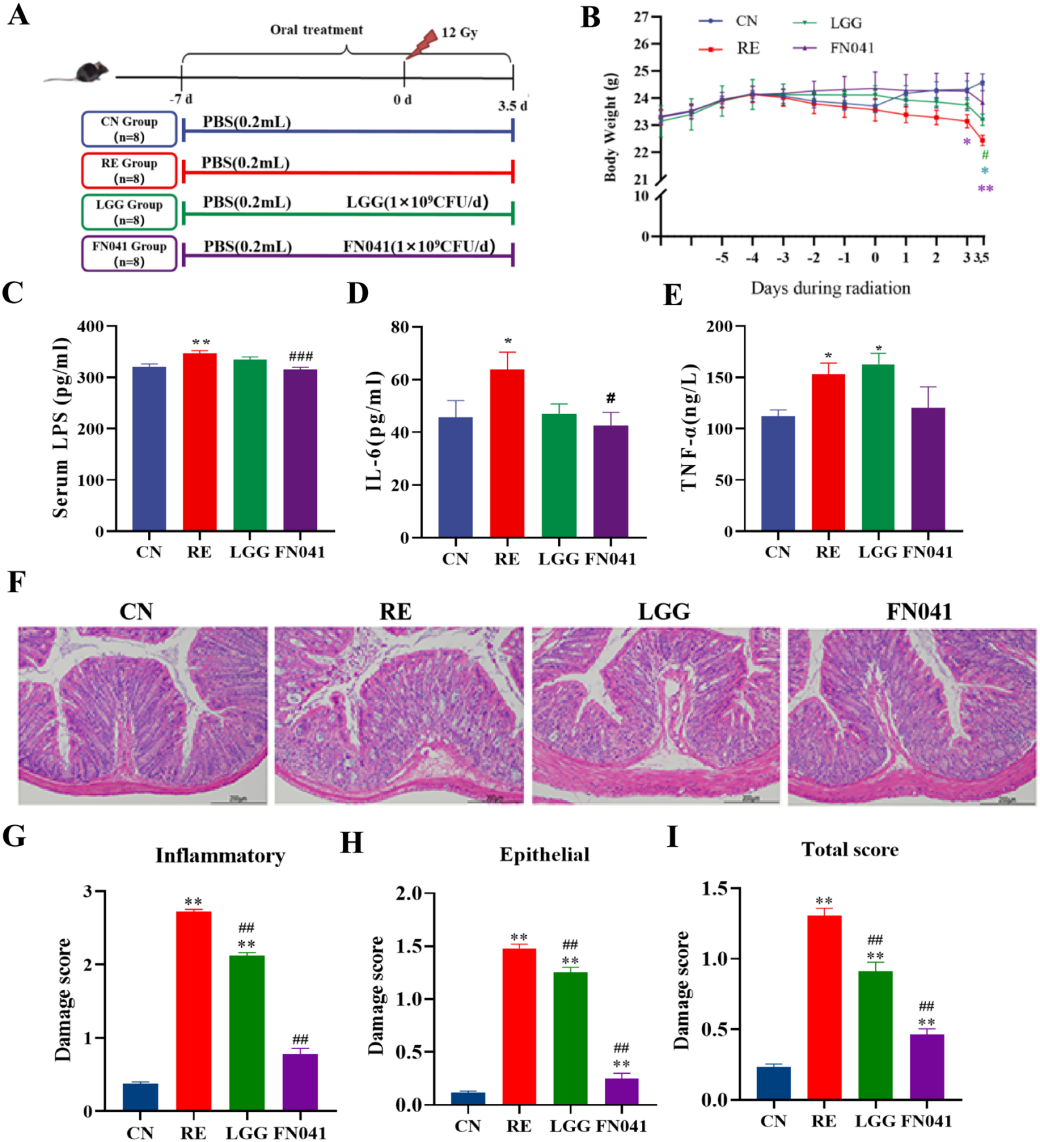
The improvement effect of *Lactobacillus reuteri* FN041 on radiation enteritis. (A) Animal treatment process. (B) Body weight change in each group (*n* = 6). (C–E) Lipopolysaccharides, IL‐6, TNF‐α of mice in each group (*n* = 6). (F) H&E‐stained colon images. (G–I) Damage score of mice in each group (*n* = 3). Data are presented as mean ± SD; *p < 0.05, **p < 0.01 as compared with the CN group; ^#^ *p* < 0.05, ^##^ *p* < 0.01 as compared with the RE group.

Mice received an abdominal x‐ray irradiation at a dose of 12 Gy and were used to establish an RE model. The animal treatment procedure is described in Figure 1A. The body weight was significantly decreased in the RE group compared to the CN group (Figure 1B). In contrast, the FN041 and LGG treatments markedly suppressed the decreased body weight after 2 days of irradiation. Moreover, the LPS level (an indicator of intestinal barrier function) in the RE group was significantly higher than that in the CN group (Figure 1C). Abdominal IR resulted in necrosis and exfoliation of intestinal epithelial cells, shallow crypts, and disordered goblet cells. The HE results showed that FN041 and LGG administration inhibited necrosis and exfoliation of intestinal epithelial cells, increased crypt depth and goblet cells (Figure 1F). In addition, the levels of inflammatory cytokines in RE mice were significantly reduced after treatment with FN041 (Figure 1D,F). In addition, the administration of FN041 and LGG significantly reduced inflammation, epithelial, and total injury scores (Figure 1G–I).

### 3.2 *Lactobacillus reuteri* FN041 Recovered the Mucus Barrier Integrity

**FIGURE 2:**
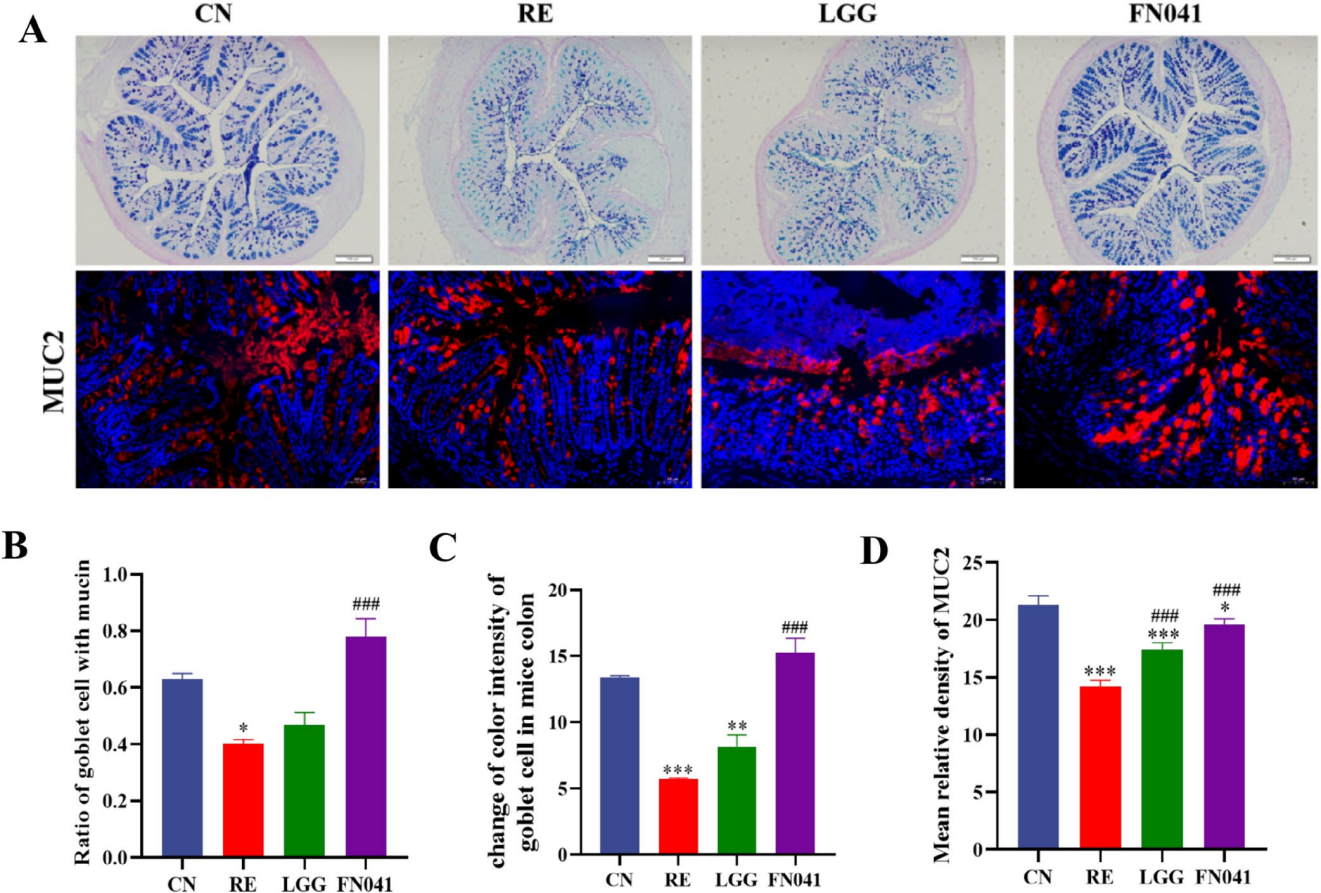
*Lactobacillus reuteri* FN041 improved mucus integrity. (A) Alcian blue staining images of colon tissues. (B) Image of immunofluorescence of Muc2. (C) Quantification of goblet cells. (D) Quantification of Muc2. Data are presented as mean ± SD; *p < 0.05, **p < 0.01, ***p < 0.001 as compared with the CN group; ^#^ *p* < 0.05, ^##^ *p* < 0.01, ^###^ *p* < 0.001 as compared with the RE group.

Mucus layers consist of mucin, which is the first line of barrier to trap mucosal aggressors such as bacteria, endogenous proteases, and secondary bile acids. These layers could be periodically replenished to sustain mucosal health and function. Mucin 2 (Muc2) is the most abundant mucin in the intestine with a typical thickness of 100–800 μm. Therefore, goblet cell numbers and Muc2 expression were measured to investigate the effect of FN041 on the mucus layer. The number of mucus‐producing cells, goblet cells, in the RE group was significantly lower than that in the CN group, while FN041 restored it (Figure 2C,D). As shown in Figure 2A, the ratio of the blue‐stained area to the total in the RE group was lower compared to the CN group. The expression of the main mucin (Muc2) in the intestine was significantly reduced after irradiation. In contrast, the administration of FN041 and LGG significantly increased the expression of Muc2 (*p* < 0.05) (Figure 2B,E). These results indicate that FN041 might recover the mucus barrier by increasing the number of goblet cells and stimulating the expression of Muc2.

### 3.3 *Lactobacillus reuteri* FN041 Restored the Physical Barrier Integrity

**FIGURE 3:**
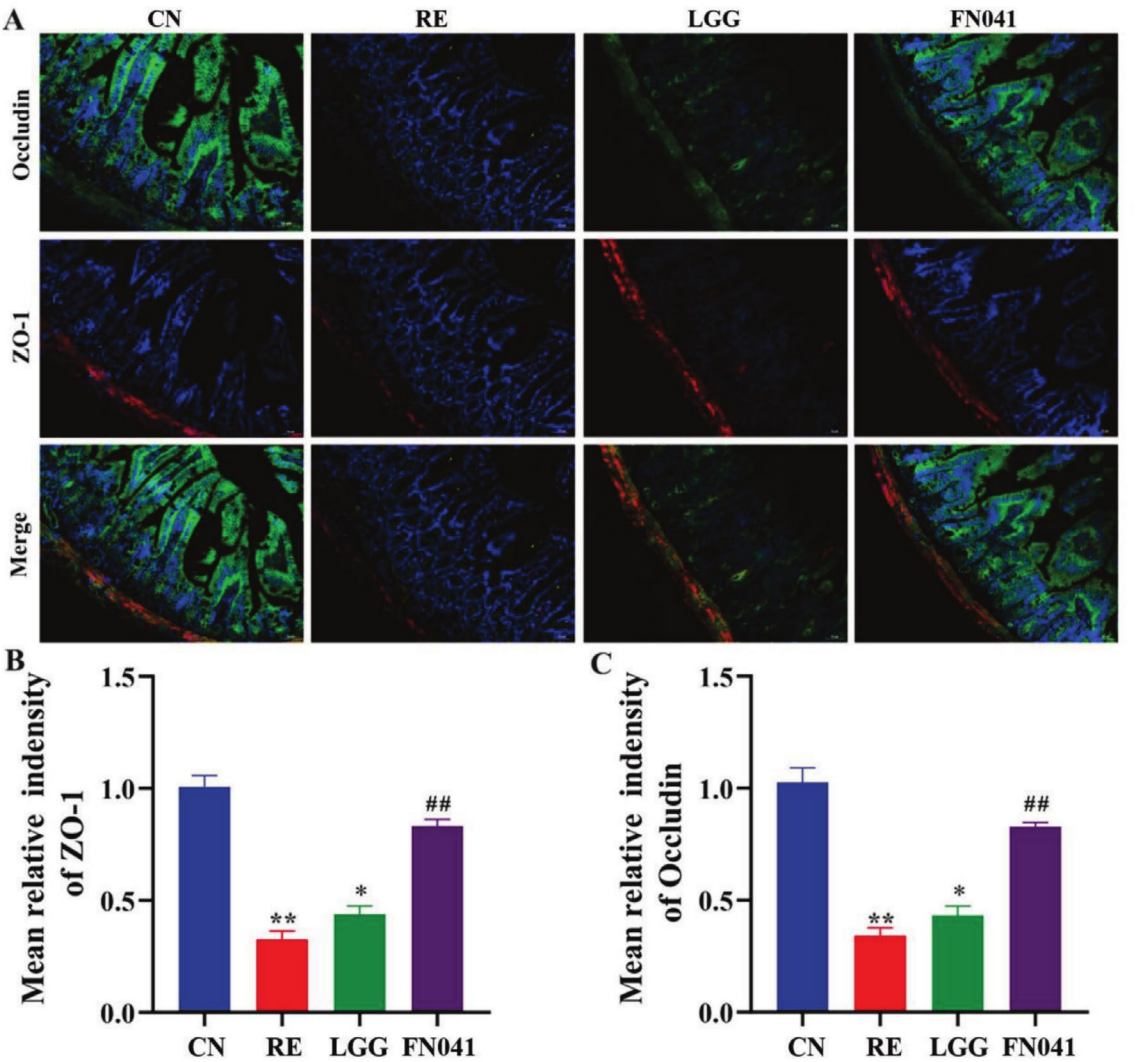
*Lactobacillus reuteri* FN041 improved physical integrity. (A) Representative images of colon tissues. (B) Quantification of ZO‐1. (C) Quantification of Occludin. Data are presented as mean ± SD (*n* = 3); *p < 0.05, **p < 0.01 as compared with the CN group; ^#^ *p* < 0.05, ^##^ *p* < 0.01 as compared with the RE group.

The specific tight junction architecture of the intestinal epithelium is the second line of the intestinal barrier. The tight junction proteins would be damaged after irradiation, which increases the intestinal bacterial translocation and endotoxin circulation. As shown in Figure 3A, the relative expression of Occludin and ZO‐1 was obviously reduced in the RE group compared to the CN group. FN041 could increase the expression of Occludin and ZO‐1. As expected, FN041 administration increased the expression of Occludin and ZO‐1 compared to the irradiated mice, while the effect of LGG was not significant (Figure 3B,C). These results demonstrate that FN041 improved the physical barrier by promoting the expression of Occludin and ZO‐1.

### 3.4 *Lactobacillus reuteri* FN041 Stimulated M2 Macrophage Polarization

**FIGURE 4:**
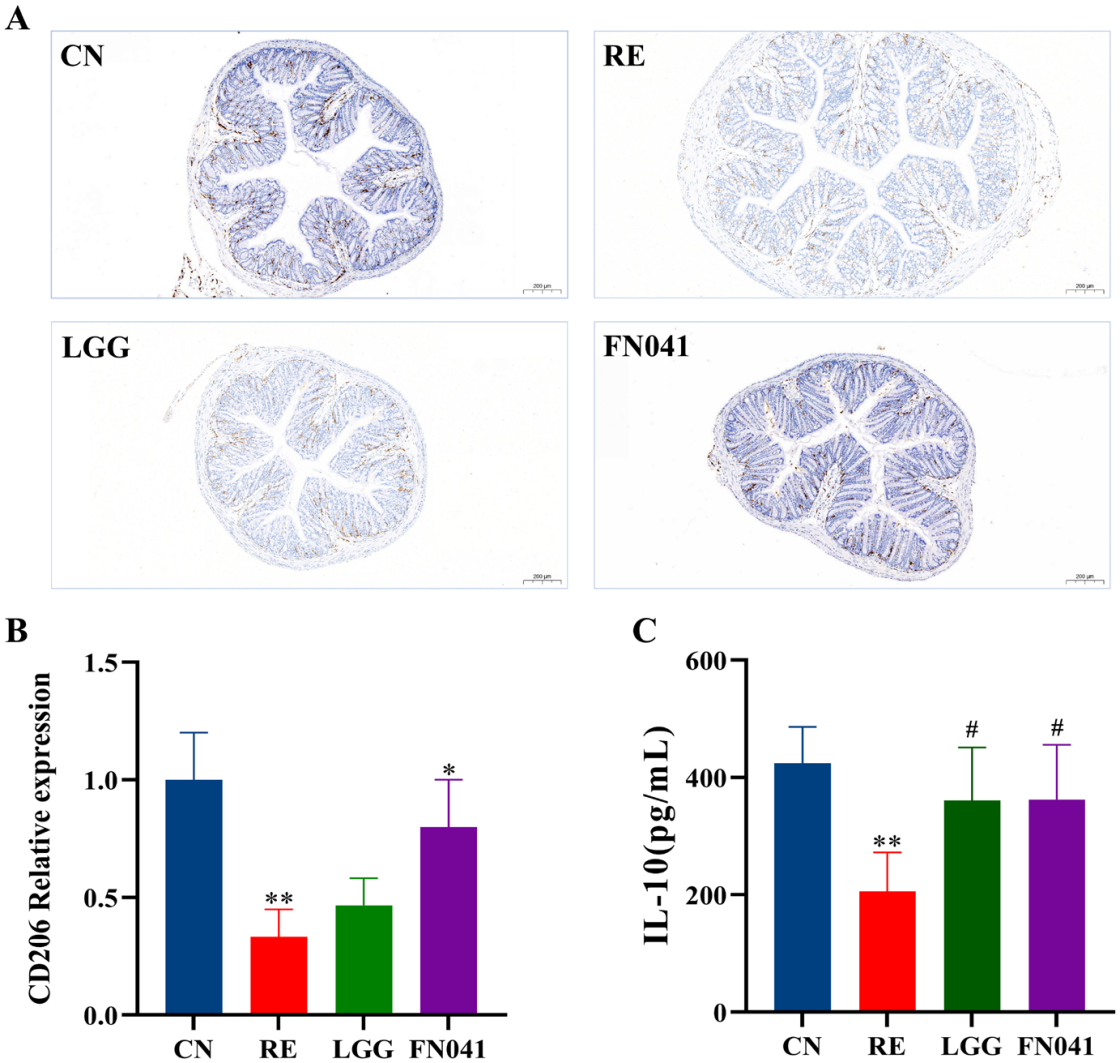
*Lactobacillus reuteri* FN041 stimulated M2 macrophage polarization. (A) Immunofluorescence staining for CD164 in colon tissues. (B) Quantification of CD206. (C) Serum levels of IL‐10. Data are presented as mean ± SD; *p < 0.05, **p < 0.01 as compared with the CN group; ^#^ *p* < 0.05 as compared with the RE group.

Macrophages are important immune cells that are rapidly recruited to damaged tissues to release inflammatory cytokines, forming an incipient inflammatory environment (Gordon and Taylor 2005). Macrophages polarize into M2 phenotypes, which can secrete anti‐inflammatory factors, inhibit inflammation, and promote tissue repair and regeneration. Hence, M2 macrophages in the colon were investigated. As expected, the M2 phenotype expression was notably suppressed after irradiation. Compared to the RE group, FN041 treatment improved the ratio of M2 phenotype in colon tissues (Figure 4A,B). Regulating immune cells could improve the secretion of pro‐inflammatory cytokines and the inflammatory response. In Figure 1D,E, the levels of TNFα and IL‐6 in the RE group were significantly enhanced compared with the CN group, whereas FN041 supplementation reversed the expression trend. In addition, IL‐10, a secretion from M2 macrophages, was also detected. As shown in Figure 4, the IL‐10 level in the RE group was significantly decreased compared with the CN group, while FN041 and LGG intervention enhanced the IL‐10 level. The above results imply that FN041 suppresses inflammatory responses by promoting M2 macrophage polarization.

### 3.5 *Lactobacillus reuteri* FN041 Regulated the Gut Microbiome and Metabolic Profiles

**FIGURE 5:**
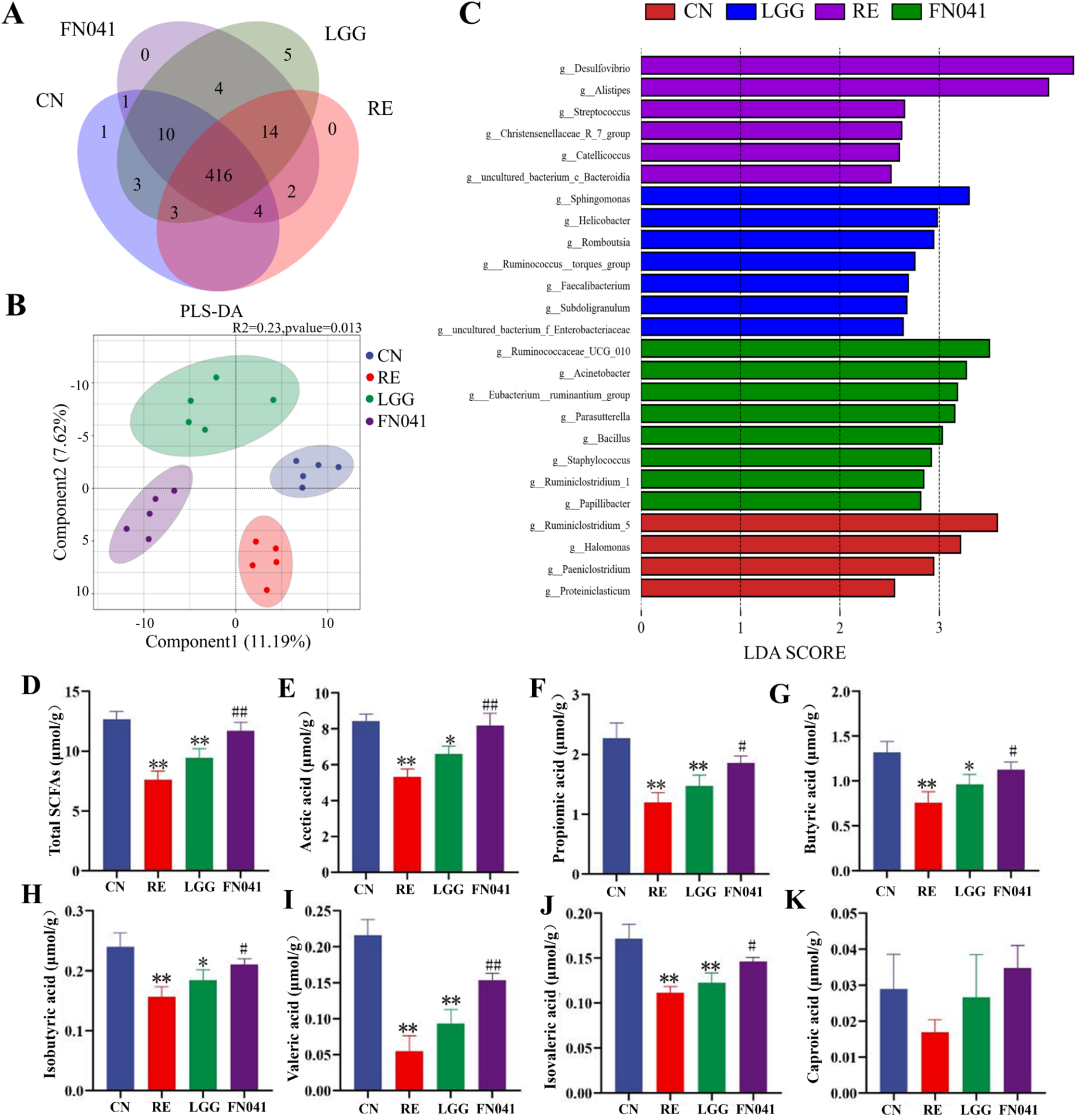
Effect of *Lactobacillus reuteri* FN041 on the gut microbiome structure in radiation enteritis mice. (A) Venn diagrams of OTUs of each group. (B) PLS‐DA results of each group. (C) Linear discriminant analysis of the microflora composition. (D) Total short‐chain fatty acids. (E) Acetic acid. (F) Propionic acid. (G) Butyric acid. (H) Isobutyric acid. (I) Valeric acid. (J) Isovaleric acid. (K) Proproic acid. Data are presented as mean ± SD; *p < 0.05, **p < 0.01 as compared with the CN group; ^#^ *p* < 0.05, ^##^ *p* < 0.01 as compared with the RE group.

It has been reported that gut microbiota dysbiosis is a vital factor in the progression of RE. The Venn diagram shows that there are 1, 0, 5, and 0 unique OTUs among the CN, RE, LGG, and FN041 groups (Figure 5A). The partial least squares discriminant analysis (PLS‐DA) result showed that the diversity of species was influenced by FN041 (Figure 5B). Compared with the CN group, the relative abundance of pathogenic bacteria such as *Desulfovibrio* and *Streptococcus* was higher in the RE group. The LGG intervention group exhibits higher relative abundances of bacterial genera such as *Sphingomonas*, *Helicobacter*, and *Romboutsia*. The relative abundance of bacterial genera including *Ruminococcaceae‐UCG‐010*, *Papillibacter*, and *Bacillus* was higher in the FN041 intervention group (Figure 5). The protective effects of FN041 on RE are also linked to a series of changes in metabolic alterations (Figure 5D–K). As shown in Figure 5E–G, the contents of beneficial metabolites (acetate, propionate, and butyrate) from bacteria were notably reduced after irradiation, while FN041 intervention reversed this abundance trend. Butyrate supplementation is effective in attenuating intestinal barrier permeability damage (Li, Lin, et al. 2021). An acetate‐yielding diet effectively improves the occurrence of intestinal infection and inflammation and significantly increases the expression of mucin 2 in the colon (Yap et al. 2021).

**FIGURE 6:**
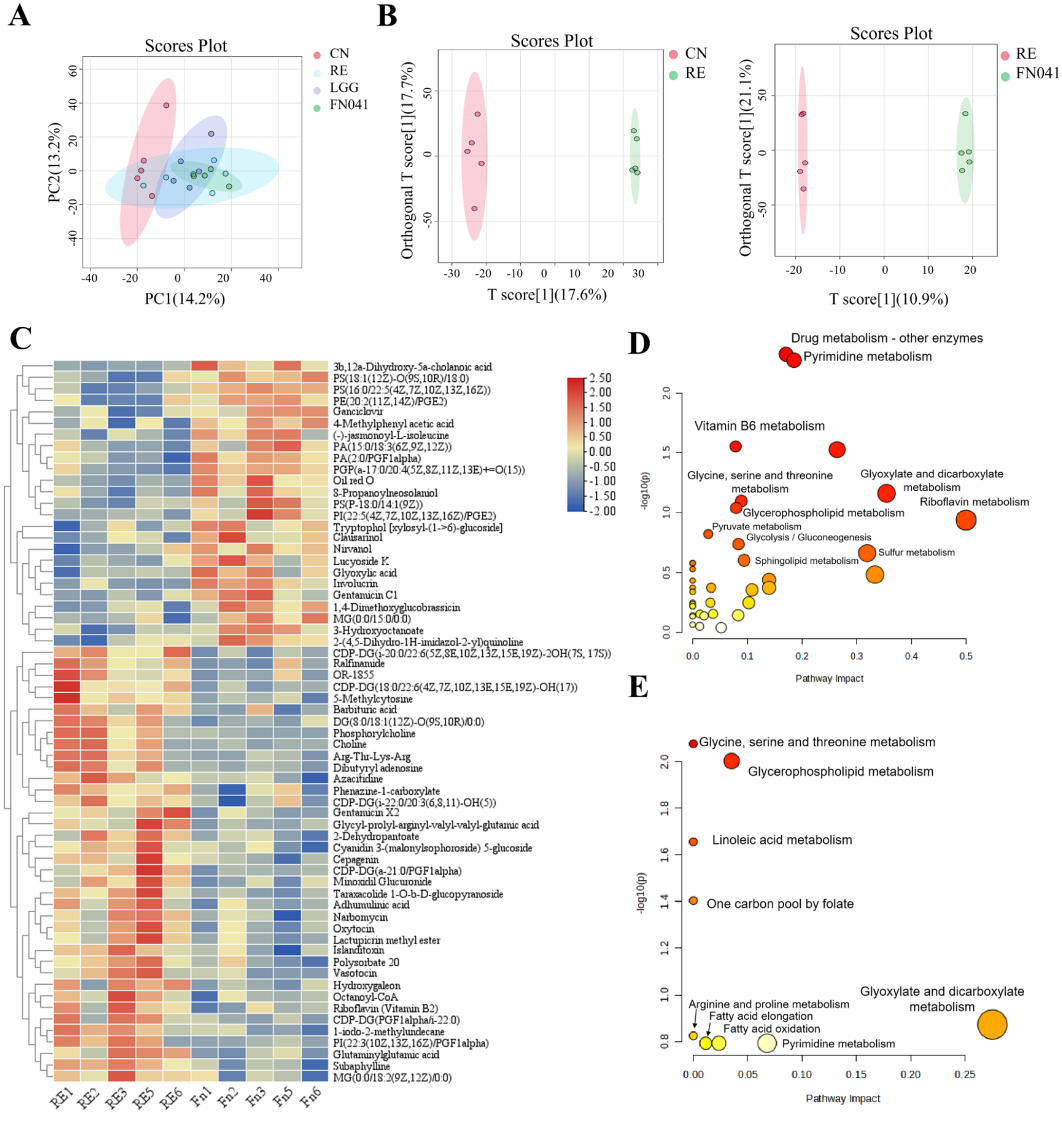
*Lactobacillus reuteri* FN041 alters metabolic profiles in radiation enteritis. (A) PCA principal component analysis. (B) Orthogonal partial least squares discriminant analysis (OPLS‐DA) of NC versus RE and RE versus FN041. (C) Heatmaps of the differential metabolites of RE versus FN041. (D) KEGG path analysis of the differential metabolites of NC versus RE. (E) KEGG path analysis of the differential metabolites of RE versus FN041.

Moreover, other metabolites were determined using untargeted metabolic profiles from serum. The individual groups showed different metabolic profiles (Figure 6A). Orthogonal partial least squares discriminant analysis (OPLS‐DA) showed that administration of FN041 significantly improved metabolite levels in mice with RE (Figure 6B). Metabolites with VIP > 1 and *p* < 0.05 were considered statistically significant. Compared with the RE group, FN041 significantly decreased 62 metabolites and increased 41 metabolites in RE mice. After FN041 treatment, the core intermediate product CDP‐DG in phospholipid synthesis was significantly downregulated, while the levels of prostaglandins and their phospholipid derivatives (including PI, PE, PA, and PS) were significantly upregulated (Figure 6C). Additionally, threonine and tryptophan‐related metabolites (L‐threoneopterin and tryptophol) were significantly higher in the FN041 group compared to the RE group. The levels of glycine and histidine‐related metabolites (glutamic acid, glutamate, glutaminyl, glutamic acid, and histidylthreonine) were decreased after FN041 treatment (Figure 6D,E). These results indicate that FN041 treatment can mediate the gut microbiota structure and metabolites.

## 4 Discussion

As a radiosensitive organ, the intestine is susceptible to injury during pelvic radiotherapy, leading to radiation‐induced intestinal injury, which has gradually increased (Gao et al. 2024). This progressive condition has few therapeutic options available and can lead to substantial long‐term morbidity and mortality. Improving intestinal barrier function is one of the core strategies for the treatment of radiation‐induced enteritis. In the present animal study, we observed that FN041 profoundly improved the physical barrier and mucus barrier. Moreover, FN041 could restructure the gut microbiota, regulate metabolites, and promote macrophage M2 polarization.

Radiation‐induced gut microbiota dysbiosis is a key mechanism responsible for the progression of radiation‐induced intestinal injury (Wang et al. 2019), which also affects tissue repair. Ionizing radiation can disrupt the gut microbiota, characterized by a decrease in beneficial bacteria (such as Lactobacillaceae and Ruminococcaceae) and an abnormal increase in potential pathogenic bacteria (such as *Akkermansia muciniphila* ) (Wang et al. 2025). Improving the gut microbiota can effectively restore the intestinal barrier and alleviate radiation‐induced intestinal damage. After fecal microbiota transplantation in germ‐free mice, there was a reduction in crypt atrophy, restoration of goblet cell numbers, decreased lymphocyte infiltration, and improvement in intestinal injury severity (Gao et al. 2024). Three patients underwent fecal microbiota transplantation and showed significant improvements in diarrhea, rectal hemorrhage, and abdominal/rectal pain (Liu et al. 2023). We found that FN041 significantly improved the gut microbiota structure of RE mice. In particular, FN041 supplementation increased the relative abundance of *Ruminococcaceae‐UCG‐010*, *Papillibacter*, and *Bacillus*. Interestingly, the gut microbiota plays a pivotal role in regulating the intestinal immune system and facilitating tissue repair (Ning et al. 2024). For instance, *Bacillus subtilis* and *Enterococcus faecium* could decrease the mortality and injury scores of cecal ligation puncture‐induced mice by suppressing the mouse ileal M1 macrophage polarization (Guo et al. 2019). *Lactobacillus intestinalis* alleviated RE by suppressing Th17 cells and inhibiting the secretion of serum amyloid A and SAA2 (Wang et al. 2023). *Lactobacillus johnsonii* mediates intestinal barrier homeostasis by promoting M2 macrophage polarization (Tao et al. 2025). In our study, FN041 enhanced macrophage polarization toward M2 and reduced levels of inflammatory factors. Promoting macrophage M2 polarization alleviates deoxynivalenol‐induced disruption of the intestinal barrier (Fan et al. 2024). Additionally, studies have found that *Lactobacillus murinus* promotes M2 polarization by inhibiting the TLR2/MyD88 pathway (Hu et al. 2022). Based on these studies, we inferred that FN041 upregulates probiotic abundance directly or indirectly to modulate macrophage M2 polarization, thus enhancing intestinal barrier function.

Accumulating evidence has confirmed that intestinal flora may also improve macrophage polarization through metabolites. It is well known that short‐chain fatty acids (SCFAs) serve as energy substrates for macrophages, promoting mitochondrial oxidative phosphorylation (OXPHOS), which is a central metabolic feature of M2 macrophages (Duan et al. 2023; Xiao et al. 2024). In our study, FN041 significantly increased the levels of short‐chain fatty acids such as acetic, propionic, and butyric acids. Fatty acid oxidation facilitates macrophage activation through bioenergetics and the production of a nuclear‐cytoplasmic pool of acetyl‐CoA for histone acetylation (Covarrubias et al. 2016). Butyric acid salts, functioning as histone deacetylase (HDAC) inhibitors, can activate the promoter regions of M2‐related genes such as IL‐10 and PPARγ, thereby enhancing their transcriptional activity. Butyric acid significantly upregulates Arg1 expression in intestinal macrophages by over threefold, leading to a marked suppression of mucosal inflammation (Ji et al. 2016). Furthermore, we observed a significant downregulation of the core intermediate product CDP‐DG in phospholipid synthesis following treatment with FN041, while levels of prostaglandins and their phospholipid derivatives, including PI, PE, PA, and PS, were markedly upregulated. Downregulation of CDP‐DG, a central intermediate in phospholipid synthesis, may reduce de novo phospholipid production, leading to the release of ATP and acetyl‐CoA to support FAO, thereby promoting M2 polarization (Nomura et al. 2016). Prostaglandins (e.g., PGE2, PGF1α) serve as key signaling molecules for M2 polarization. Upon binding with PI, PE, PA, or PS, their function is enhanced to provide sustained, precise, and synergistic anti‐inflammatory signals, directly promoting the establishment of the M2 phenotype (Santiso et al. 2024). In conclusion, FN041 may promote M2 polarization of macrophages, enhance intestinal barrier repair, and ameliorate radiation‐induced intestinal injury through modulation of the gut microbiota and its metabolites.

This study also has certain limitations. Studies suggest that FN041 drives M2 polarization of macrophages by modulating gut microbiota metabolites, but it remains unclear how these metabolites target macrophages. Future studies should complement microbiota transplantation (FMT) or metabolite intervention experiments to validate causality.

## 5 Conclusion

In conclusion, we evaluated the protective effect of FN041 on colonic damage and inflammation induced by radiotherapy. The results suggested that FN041 treatment improved colon histology, physical barrier integrity, and mucus integrity. In addition, FN041 suppresses the inflammatory response and enhances M2 macrophage polarization. The possible mechanism was related to the alteration of gut microbiota and its metabolites. The research uncovers the critical role of gut microbiota and its metabolites in alleviating intestinal damage and providing a novel and effective therapy in RE.

## Author Contributions

**Suisui Jiang:** conceptualization (equal), formal analysis (equal), writing – original draft (equal), writing – review and editing (equal). **Sai Yao:** conceptualization (equal), data curation (equal), formal analysis (equal), supervision (equal), writing – original draft (supporting), writing – review and editing (equal). **Chunyun Lu:** data curation (equal), investigation (equal). **Ying Zhou:** data curation (equal), investigation (equal). **Tianlin Gao:** conceptualization (equal), data curation (equal), resources (equal). **Shichao Liu:** funding acquisition (equal), project administration (equal), resources (equal), supervision (lead), writing – review and editing (equal). **Feng Zhong:** funding acquisition (equal), project administration (equal), resources (equal), supervision (lead), writing – review and editing (equal).

## Ethics Statement

All procedures were performed in accordance with the Guidelines in the Care and Use of Animals and with the approval of the Ethical Committee of Experimental Animal Welfare of Qingdao University (no. 20200924C575020210118046).

## Conflicts of Interest

The authors declare no conflicts of interest.

## Data Availability

The data that support the findings of this study are available from the corresponding author upon reasonable request.
